# A case of severe nephrotoxicity associated with long-term dietary supplement use

**DOI:** 10.5414/CNCS109180

**Published:** 2017-08-02

**Authors:** Farrukh M. Koraishy, Gilbert W. Moeckel, David S. Geller

**Affiliations:** 1 Section of Nephrology, Department of Internal Medicine, Saint Louis University, MO,; 2 Renal Section, Saint Louis VA Hospital, MO,; 3 Department of Pathology,; 4 Section of Nephrology, Department of Internal Medicine, Yale University, CT, and; 5 Section of Nephrology, West Haven VA Hospital, CT, USA

**Keywords:** dietary supplement, nephrotoxicity, membranous nephropathy, interstitial nephritis, tubular necrosis, cherry extract

## Abstract

Dietary supplements are widely used for their perceived health benefits without side effects and hence have minimal regulation. However, they have been associated with various toxicities including kidney disease. We report a 65-year-old male who had very heavy daily intake of dietary supplements for 3 years. He presented with acute kidney injury and nephrotic-range proteinuria. The renal biopsy showed acute tubular necrosis with vacuolization, acute interstitial nephritis, and secondary membranous nephropathy, consistent with an non-steroidal anti-inflammatory drug (NSAID)-like nephropathy. This was postulated to be related to the cyclooxygenase (COX) inhibitors (anthocyanins) in cherry extract that was a significant part of the patient’s dietary supplement use. His proteinuria completely resolved and serum creatinine stabilized after discontinuation of all dietary supplements and a prolonged (5 months) course of prednisone. Clinicians are advised to specifically inquire about dietary supplements, especially cherry extract, as a potential cause of new-onset renal failure and proteinuria.

## Introduction 

Complementary and alternative medicine (CAM) is widely practiced all over the world [1]. “Nutraceuticals” constitute a multi-billion dollar industry, and include a range of products composed of isolated nutrients, dietary supplements, herbal products, specific diets, functional foods, and processed foods [2]. A dietary supplement is a form of nutraceutical that contains nutrients derived from food products that are concentrated in liquid, gel, or capsule form. They are considered by the US Food and Drug Administration (FDA) as a “food” and not as “pharmaceutical drugs”, hence manufacturers do not require FDA approval before marketing [3]. The various label claims associated with these products have a variable level of regulation [4]. While dietary supplements are advertised and consumed with the promise of providing significant health benefits without side effects, evidence suggests minimal benefits and potential harm to human health [5, 6, 7, 8]. In addition to damage to other organs [9], a diverse range of nephrotoxicities have been reported with dietary supplements [10, 11]. Despite these safety concerns, a significant number of patients do not report dietary supplement use, and physicians often do not inquire about them [12]. 

## Case report 

A 65-year-old white male with a history of hypertension, borderline diabetes type 2, and hypothyroidism was referred for nephrotic range proteinuria (16.6 g/day), anasarca, and fatigue with normal serum creatinine (SCr) (1.1 mg/dL), serum albumin (SAlb), and thyroid function tests. Previous labs on chart review showed a normal SCr of 1.0 mg/dL 3 months ago. There were no recent urine studies except a urine dipstick negative for protein from 20 years ago. Review of systems was otherwise negative, and the urine sediment was bland. His medications included levothyroxine, amlodipine, and chlorthalidone. He denied the use of non-steroidal anti-inflammatory drugs (NSAIDs) and herbs, but he did report taking numerous over-the-counter (OTC) dietary supplements for aging, joint pains, energy, and sexual performance. He mentioned that he started taking all the supplements 3 years ago on the recommendation of his chiropractor (Table 1). A serologic workup for secondary causes of glomerulonephritis (GN) was negative. Benazepril and simvastatin were added to his medication regimen. He was advised to stop all OTC supplements but was lost to follow-up. 

He returned 7 months later when his SCr had increased to 2.3 gm/dL with persistent proteinuria. His SAlb was down to 2.2 g/dL; his urine now evidenced white cells, epithelial cells, and granular casts. He had continued to take the items listed in Table 1 but denied other agents. He was again advised to stop all nonprescription drugs and after extensive counselling, he agreed. A repeat workup for secondary causes of GN was negative. The proteinuria and azotemia continued for 2 weeks off the supplements at which point a renal biopsy was performed. 

**Figure 1.**
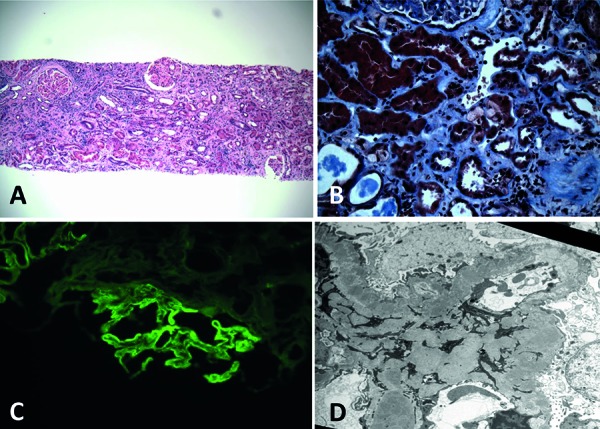
A: Acute interstitial nephritis (H & E, 100×). B: abnormal tubular cytoplasmic vacuoles (Trichrome, 400×). C: IF: granular IgG staining along glomerular basement membranes. D: EM: numerous sub epithelial and mesangial immune complex deposits (5,000×).

The renal biopsy showed severe acute tubular necrosis (ATN) with prominent vacuoles in the tubular cell cytoplasm and extensive lymphocytic infiltrates with occasional eosinophils and tubulitis consistent with acute interstitial nephritis (AIN) (Figure 1A, B). There was chronic inflammation and significant scarring with 40% tubulointerstitial fibrosis. The immunofluorescence (IF) showed linear IgG staining (Figure 1C), and electron microscopy (EM) showed subepithelial and mesangial immune complex deposits (Figure 1D), consistent with secondary membranous nephropathy (MN). While AIN and vacuolar ATN could have been due to many of the dietary supplements (Table 1), the association of AIN with nephrotic range proteinuria is classically reported with NSAIDs [13]. Hence, our provisional diagnosis was renal damage from the cherry extract, a significant component of the supplements, which has NSAID-like properties [14, 15]. 

First-line treatment for nephrotoxic renal disease is discontinuation of the offending agent, but high-dose steroids have been reported to be beneficial in cyclooxygenase (COX) inhibitor-related proteinuric AIN [16]. In light of the persistent azotemia and proteinuria 3 weeks after supplement discontinuation, immunosuppressive therapy (prednisone 1.0 mg/kg/day) was initiated. After 5 weeks, SCr declined to 1.6 mg/dL with reduced but still nephrotic range proteinuria (spot urine protein/creatinine ratio (UPCR) = 7.8 g/g) and a bland urine sediment. He remained asymptomatic during a gradual steroid taper with a stable SCr and declining proteinuria (UPCR = 1.4 g/g). Two weeks after stopping prednisone, however, edema, nephrotic proteinuria (UPCR = 4.6 g/g), and azotemia SCr 2.6 mg/dL) recurred. He denied new medications, including previous OTC drugs. Urine sediment again showed granular casts with occasional epithelial cells. A repeat renal biopsy showed similar (although less “active”) findings to the first biopsy. Since COX inhibitor-related AIN has been reported to sometimes require a prolonged steroid course [16], the patient was treated with 1.0 mg/kg daily prednisone for another 10 weeks. His SCr remained stable at 2.1 – 2.3 mg/dL and proteinuria remitted (UPCR = 0.03 g/g). Overall, he received a total of 5 months of steroid therapy. 

Now after 3 years off prednisone (and the OTC supplements) and with continued treatment with statins, ACE inhibitors, and strict blood pressure control, his proteinuria remains low grade (UPCR < 50 mg/g) and SCr remains stable in the 2.2 – 2.5 mg/dL range. 

## Discussion 

Dietary supplements are very widely used by the public for perceived health benefits but some of them have been associated with kidney disease [10, 11]. Here we report a patient with nephrotoxic ATN and AIN with severe proteinuria (secondary MN) after many years of heavy consumption of dietary supplements, which resolved after prolonged cessation of the supplements and 5 months of high-dose steroid therapy. 

The patient’s clinical course is strongly suggestive of a toxic injury caused by the OTC supplements he was taking. A detailed study of the ingredients in each supplement (Table 1) showed reported nephrotoxicity associated with the following compounds: cinnamon oil [17], vitamins A, D, and E [18], folic acid [19], calcium [20], iodine [21], copper [22], molybdenum [23], sweet cherry fruit [14], rhizome extract [24], and vanadium [25]. Of these, the highest proportional amount was of “sweet cherry extract” (109 mg per serving), which is known to contain COX inhibitors (anthocyanins) that are responsible for its anti-inflammatory NSAID-like effects [15, 26]. 

The combination of AIN with nephrotic-range proteinuria is highly suggestive of COX inhibitor-related renal damage. Our patient’s cumulative intake of cherry extract was estimated at over 200 g over 3 years. Given the known NSAID-like activity in cherry extract [15], the typical pathologic findings and finally the classical and clinical course of the patient (complete resolution of proteinuria and halt of further renal decline after discontinuation of the agents and a prolonged steroid course), we believe the cherry extract was most likely responsible for the observed nephropathy. 

Cherry extract is a commonly used dietary supplement for anti-oxidant and anti-inflammatory purposes and is reported to have no side effects [26, 27]. Hemodynamic acute kidney injury (AKI) has been reported in a patient consuming excessive cherry concentrate [14] for gout. Recent studies have shown that flavonoids and especially anthocyanins, present in high concentrations in cherry extract, inhibit both COX 1 and 2 at levels similar to that of conventional NSAIDs [15, 28]. NSAIDs are well known to be associated with ATN, AIN, nephrotic syndrome, and chronic kidney disease (CKD) [29]. As the pathogenesis of NSAID-induced nephritis is believed to be due to COX inhibition [13, 30], we think it likely that cherry extract could produce a similar clinical phenotype. Clinical data also support our belief that the nephropathy was due to a COX-inhibitory effect. Our case fulfilled all the criteria for NSAID-induced secondary MN in a large retrospective study of patients with early MN [31], including exclusion of secondary causes of MN, onset of nephrotic syndrome while on non-toxic NSAID doses, and remission off the drug. Unlike other drugs, the interstitial nephritis reported with NSAIDs is often associated with nephrotic-range proteinuria with a lesion consistent with secondary MN [13, 32, 33], minimal change disease [13, 33, 34], or focal sclerosis [13]. Additionally, the classical picture of an allergic reaction that includes fever, rash, and eosinophilia is typically absent in NSAID-associated nephritis as it was here, likely due to the anti-inflammatory nature of the agent [35]. We did not obtain IgG4 or phospholipase A2 receptor (PLA2R) testing in our patient. While a positive stain for IgG4 and PLA2R is considered to be relatively specific for primary/idiopathic MN, both stains were reported to be present in NSAID-induced secondary MN [36]. Hence, the presence or absence of IgG4 or PLA2R staining would not have necessarily aided in the diagnosis. 

There is no consensus on the optimal treatment of NSAID-induced nephritis. Steroids have long been proposed for interstitial nephritis in patients with no improvement after the removal of the potential offending agent [37, 38], although the optimal dose and duration is not established. In NSAID-induced nephritis, while most cases resolve after discontinuation of the drug [39], treatment with steroids is sometimes required [16, 40]. In our patient, a lengthy steroid course was required. Persistence of chronic kidney disease despite resolution of proteinuria after NSAID discontinuation has been reported previously [41]. Among AIN induced by drugs, NSAIDs are the most likely to lead to permanent renal damage [42]. The patient has been extensively educated that he is likely to be predisposed to recurrent nephropathy from dietary supplements with subsequent exposures [43]. 

To our knowledge, this is the first report of an extensive glomerular and tubulointerstitial injury associated with dietary supplements. Clinicians should be aware of its possibility and potential management of a NSAID-like nephrotoxicity likely from cherry extract and should always ask patients about their use of nutraceuticals and dietary supplements. Most patients do not consider these as medicines and will not mention them unless specifically inquired. It is also important for clinicians to report these cases to increase awareness amongst healthcare providers and the public [44]. 

## Conflict of interest 

None to declare. 

**Table 1.** List of dietary supplements used by the patient.

Dietary supplement	Active ingredients and potentially nephrotoxicity based on published literature (check reference)
Nitric balance (apex energetics)	• ATP • Xanthinol nicotinate • N-acetyl L-carnitine • Huperzine A • α-GPC • Vinpocetine • Filtered water • Vegetable glycerin • Honey • Evening primrose oil • Xanthan gum • Luo han guo fruit extract • Gluconic acid • Sodium citrate • Cinnamon oil (potentially nephrotoxic [17]) • Natural flavor • Potassium sorbate • Stevia extract • Citric acid
Clinical nutrients for men (integrative therapeutics)	• Dietary fiber • Vitamin A (potentially nephrotoxic [18]) • Vitamin C • Vitamin D (potentially nephrotoxic [18]) • Vitamin E (potentially nephrotoxic [18]) • Vitamin K • Thiamin • Riboflavin • Niacin • Vitamin B_6 _ • Folic acid (potentially nephrotoxic [19]) • Vitamin B_12 _ • Biotin • Pantothenic acid • Calcium (potentially nephrotoxic [20]) • Iodine (potentially nephrotoxic [21]) • Magnesium • Zinc • Selenium • Copper (potentially nephrotoxic [22]) • Manganese • Molybdenum (potentially nephrotoxic [23]) • Sodium • Potassium • Choline bitartrate • Cinnamon bark extract • Proprietary blend: - Sweet cherry fruit (potentially nephrotoxic [14]) - Green tea leaf extract - Grape seed extract - Pomegranate fruit extract - Giant knotweed root - Rhizome extract (potentially nephrotoxic [24]) • Ginger (*Zingiber officinale*) rhizome extract • Vegetable blend: broccoli, cabbage, carrot, collard greens, radish sprouts, tomato, mustard greens, kale, spinach • Inositol • Maca (*Lepidium meyenii*) root extract • Asian (*Panax ginseng*) ginseng root extract • Bilberry (*Vaccinium myrtillus*) fruit extract • Boron (as sodium borate) • Lycopene • Lutein • Vanadium (potentially nephrotoxic [25]) • Zeaxanthin
Detox antiox (designs for health)	• Vitamin C • Vitamin E • Biotin • Zinc • Selenium • Manganese • Molybdenum (potentially nephrotoxic [23]) • N-acetyl cysteine • Leucine • α lipoic acid • Green tea • Turmeric extract • Grape seed extract

## References

[1] Harris PE Cooper KL Relton C Thomas KJ Prevalence of complementary and alternative medicine (CAM) use by the general population: a systematic review and update. Int J Clin Pract. 2012; 66: 924–939. 22994327 10.1111/j.1742-1241.2012.02945.x

[2] Kalra EK Nutraceutical – definition and introduction. AAPS PharmSci. 2003; 5: E25. 14621960 10.1208/ps050325 PMC2750935

[3] Are dietary supplements approved by FDA? http://www.fda.gov/AboutFDA/Transparency/Basics/ucm194344.htm.

[4] Turner RE Degnan FH Archer DL Label claims for foods and supplements: a review of the regulations. Nutr Clin Pract. 2005; 20: 21–32. 16207644 10.1177/011542650502000121

[5] Guallar E Stranges S Mulrow C Appel LJ Miller ER Enough is enough: Stop wasting money on vitamin and mineral supplements. Ann Intern Med. 2013; 159: 850–851. 24490268 10.7326/0003-4819-159-12-201312170-00011

[6] Fortmann SP Burda BU Senger CA Lin JS Whitlock EP Vitamin and mineral supplements in the primary prevention of cardiovascular disease and cancer: An updated systematic evidence review for the U.S. Preventive Services Task Force. Ann Intern Med. 2013; 159: 824–834. 24217421 10.7326/0003-4819-159-12-201312170-00729

[7] Bjelakovic G Nikolova D Gluud LL Simonetti RG Gluud C Mortality in randomized trials of antioxidant supplements for primary and secondary prevention: systematic review and meta-analysis. JAMA. 2007; 297: 842–857. 17327526 10.1001/jama.297.8.842

[8] Lambert JD Sang S Yang CS Possible controversy over dietary polyphenols: benefits vs risks. Chem Res Toxicol. 2007; 20: 583–585. 17362033 10.1021/tx7000515

[9] Phua DH Zosel A Heard K Dietary supplements and herbal medicine toxicities-when to anticipate them and how to manage them. Int J Emerg Med. 2009; 2: 69–76. 20157447 10.1007/s12245-009-0105-z PMC2700222

[10] Gabardi S Munz K Ulbricht C A review of dietary supplement-induced renal dysfunction. Clin J Am Soc Nephrol. 2007; 2: 757–765. 17699493 10.2215/CJN.00500107

[11] Luyckx VA Nephrotoxicity of alternative medicine practice. Adv Chronic Kidney Dis. 2012; 19: 129–141. 22578672 10.1053/j.ackd.2012.04.005

[12] Trivedi R Salvo MC Utilization and Safety of Common Over-the-Counter Dietary/Nutritional Supplements, Herbal Agents, and Homeopathic Compounds for Disease Prevention. Med Clin North Am. 2016; 100: 1089–1099. 27542428 10.1016/j.mcna.2016.04.017

[13] Ravnskov U Glomerular, tubular and interstitial nephritis associated with non-steroidal antiinflammatory drugs. Evidence of a common mechanism. Br J Clin Pharmacol. 1999; 47: 203–210. 10190656 10.1046/j.1365-2125.1999.00869.x PMC2014171

[14] Luciano RL Acute kidney injury from cherry concentrate in a patient with CKD. Am J Kidney Dis. 2014; 63: 503–505. 24290246 10.1053/j.ajkd.2013.09.021

[15] Seeram NP Momin RA Nair MG Bourquin LD Cyclooxygenase inhibitory and antioxidant cyanidin glycosides in cherries and berries. Phytomedicine. 2001; 8: 362–369. 11695879 10.1078/0944-7113-00053

[16] Révai T Harmos G Nephrotic syndrome and acute interstitial nephritis associated with the use of diclofenac. Wien Klin Wochenschr. 1999; 111: 523–524. 10444806

[17] Koci J Jeffery B Riviere JE Monteiro-Riviere NA In vitro safety assessment of food ingredients in canine renal proximal tubule cells. Toxicol In Vitro. 2015; 29: 289–298. 25458622 10.1016/j.tiv.2014.11.002

[18] De Francesco Daher E Mesquita Martiniano LV Lopes Lima LL Viana Leite Filho NC de Oliveira Souza LE Duarte Fernandes PH da Silva SL da Silva GB Acute kidney injury due to excessive and prolonged intramuscular injection of veterinary supplements containing vitamins A, D and E: A series of 16 cases. Nefrologia. 2017; 37: 61–67. 27577045 10.1016/j.nefro.2016.05.017

[19] Stallons LJ Whitaker RM Schnellmann RG Suppressed mitochondrial biogenesis in folic acid-induced acute kidney injury and early fibrosis. Toxicol Lett. 2014; 224: 326–332. 24275386 10.1016/j.toxlet.2013.11.014 PMC3987699

[20] Narula S Tandon S Singh SK Tandon C Kidney stone matrix proteins ameliorate calcium oxalate monohydrate induced apoptotic injury to renal epithelial cells. Life Sci. 2016; 164: 23–30. 27593572 10.1016/j.lfs.2016.08.026

[21] Perrin T Hemett OM Menth M Descombes E Contrast-induced acute kidney injury following iodine opacification other than by intravascular injection. Clin Kidney J. 2012; 5: 456–458. 24175084 10.1093/ckj/sfs102 PMC3811973

[22] Dash SC Copper sulphate poisoning and acute renal failure. Int J Artif Organs. 1989; 12: 610. 2807586

[23] Bompart G Pécher C Prévot D Girolami JP Mild renal failure induced by subchronic exposure to molybdenum: urinary kallikrein excretion as a marker of distal tubular effect. Toxicol Lett. 1990; 52: 293–300. 1975134 10.1016/0378-4274(90)90039-o

[24] Wojcikowski K Wohlmuth H Johnson DW Gobe G Dioscorea villosa (wild yam) induces chronic kidney injury via pro-fibrotic pathways. Food Chem Toxicol. 2008; 46: 3122–3131. 18662738 10.1016/j.fct.2008.06.090

[25] Liu J Cui H Liu X Peng X Deng J Zuo Z Cui W Deng Y Wang K Dietary high vanadium causes oxidative damage-induced renal and hepatic toxicity in broilers. Biol Trace Elem Res. 2012; 145: 189–200. 21882068 10.1007/s12011-011-9185-8

[26] Bell PG McHugh MP Stevenson E Howatson G The role of cherries in exercise and health. Scand J Med Sci Sports. 2014; 24: 477–490. 23710994 10.1111/sms.12085

[27] Cherry fruit extract. http://www.wholehealthchicago.com/466/cherry-fruit-extract/.

[28] Vane JR Botting RM Mechanism of action of nonsteroidal anti-inflammatory drugs. Am J Med. 1998; 104: 2S–8S. 10.1016/s0002-9343(97)00203-9 9572314

[29] Clive DM Stoff JS Renal syndromes associated with nonsteroidal antiinflammatory drugs. N Engl J Med. 1984; 310: 563–572. 6363936 10.1056/NEJM198403013100905

[30] Bender WL Whelton A Beschorner WE Darwish MO Hall-Craggs M Solez K Interstitial nephritis, proteinuria, and renal failure caused by nonsteroidal anti-inflammatory drugs. Immunologic characterization of the inflammatory infiltrate. Am J Med. 1984; 76: 1006–1012. 6375363 10.1016/0002-9343(84)90849-0

[31] Radford MG Holley KE Grande JP Larson TS Wagoner RD Donadio JV McCarthy JT Reversible membranous nephropathy associated with the use of nonsteroidal anti-inflammatory drugs. JAMA. 1996; 276: 466–469. 8691554

[32] Marasco WA Gikas PW Azziz-Baumgartner R Hyzy R Eldredge CJ Stross J Ibuprofen-associated renal dysfunction. Pathophysiologic mechanisms of acute renal failure, hyperkalemia, tubular necrosis, and proteinuria. Arch Intern Med. 1987; 147: 2107–2116. 3689062 10.1001/archinte.147.12.2107

[33] Champion de Crespigny PJ Becker GJ Ihle BU Walter NM Wright CA Kincaid-Smith P Renal failure and nephrotic syndrome associated with sulindac. Clin Nephrol. 1988; 30: 52–55. 3208459

[34] Feinfeld DA Olesnicky L Pirani CL Appel GB Nephrotic syndrome associated with use of the nonsteroidal anti-inflammatory drugs. Case report and review of the literature. Nephron. 1984; 37: 174–179. 6738768 10.1159/000183239

[35] Brezin JH Katz SM Schwartz AB Chinitz JL Reversible renal failure and nephrotic syndrome associated with nonsteroidal anti-inflammatory drugs. N Engl J Med. 1979; 301: 1271–1273. 503131 10.1056/NEJM197912063012306

[36] Nawaz FA Larsen CP Troxell ML Membranous nephropathy and nonsteroidal anti-inflammatory agents. Am J Kidney Dis. 2013; 62: 1012–1017. 23773370 10.1053/j.ajkd.2013.03.045

[37] Neilson EG Pathogenesis and therapy of interstitial nephritis. Kidney Int. 1989; 35: 1257–1270. 2770107 10.1038/ki.1989.118

[38] González E Gutiérrez E Galeano C Chevia C de Sequera P Bernis C Parra EG Delgado R Sanz M Ortiz M Goicoechea M Quereda C Olea T Bouarich H Hernández Y Segovia B Praga M Early steroid treatment improves the recovery of renal function in patients with drug-induced acute interstitial nephritis. Kidney Int. 2008; 73: 940–946. 18185501 10.1038/sj.ki.5002776

[39] Markowitz GS Falkowitz DC Isom R Zaki M Imaizumi S Appel GB D’Agati VD Membranous glomerulopathy and acute interstitial nephritis following treatment with celecoxib. Clin Nephrol. 2003; 59: 137–142. 12608557 10.5414/cnp59137

[40] Esteve JB Launay-Vacher V Brocheriou I Grimaldi A Izzedine H COX-2 inhibitors and acute interstitial nephritis: case report and review of the literature. Clin Nephrol. 2005; 63: 385–389. 15909599 10.5414/cnp63385

[41] Almansori M Kovithavongs T Qarni MU Cyclooxygenase-2 inhibitor-associated minimal-change disease. Clin Nephrol. 2005; 63: 381–384. 15909598 10.5414/cnp63381

[42] Schwarz A Krause PH Kunzendorf U Keller F Distler A The outcome of acute interstitial nephritis: risk factors for the transition from acute to chronic interstitial nephritis. Clin Nephrol. 2000; 54: 179–190. 11020015

[43] Mohammed EP Stevens JM Recurrence of Arthrotec-associated nephrotic syndrome with re-challenge. Clin Nephrol. 2000; 53: 483–485. 10879670

[44] Glisson JK Walker LA How physicians should evaluate dietary supplements. Am J Med. 2010; 123: 577–582. 20493463 10.1016/j.amjmed.2009.10.017

